# Speech characteristics that differentiate stuttering and cluttering in Japanese speakers

**DOI:** 10.3389/fpsyg.2024.1408929

**Published:** 2024-11-14

**Authors:** Shuta Tomisato, Takanori Mori, Kazumi Asano, Daichi Iimura, Yasuto Yada, Saburo Takahashi, Koichiro Wasano, Takeyuki Kono, Hiroyuki Ozawa

**Affiliations:** ^1^Department of Otorhinolaryngology, Head, and Neck Surgery, Keio University School of Medicine, Shinjuku, Tokyo, Japan; ^2^Institute of Human Sciences, University of Tsukuba, Ibaraki, Japan; ^3^Department of Language Sciences, Tokyo Metropolitan University, Hachioji, Tokyo, Japan; ^4^Sumiyoshi Elementary School, Tokyo, Japan; ^5^Department of Otolaryngology, Head, and Neck Surgery, Tokai University School of Medicine, Isehara, Japan

**Keywords:** stuttering, cluttering, differential diagnosis, Japanese speaker, articulatory rate

## Abstract

**Background:**

Cluttering is a speech disorder distinct from stuttering. Despite this distinction, there is no established method to clearly differentiate the two disorders. This study aimed to use objective criteria to differentiate cluttering from stuttering in Japanese speakers.

**Methods:**

Participants were 32 consecutive native-Japanese speakers who visited the Keio University Hospital between July 2020 and January 2023 with a chief complaint of speech disfluency. One physician and two speech-language-hearing therapists concurred on a stuttering or cluttering diagnosis of the 32 patients based on recordings of the Kitsuon kensa-ho test. The frequencies of stuttering-like disfluencies (SDF) and normal disfluencies (NDF) were calculated from the Kitsuon kensa-ho, and the ratio of disfluencies (RDF) was calculated as the ratio of SDF to NDF. Differences between the cluttering and stuttering groups in the RDF and the mean articulatory rate (MAR) for oral reading and a monologue task were tested using the Mann–Whitney U test. ROC curves were used to determine the sensitivity and specificity that well-distinguished subjects with cluttering from those with stuttering; the experts’ diagnosis was the gold standard.

**Results:**

Of the 32 participants, 12 (38%) were diagnosed with cluttering and 20 (62%) with stuttering. The cluttering and stuttering groups were comparable in demographic characteristics. The RDF on monologue task had the highest sensitivity in diagnosing cluttering, and the MAR on monologue task had the highest specificity. Adopting provisional criteria of a monologue RDF greater than 1.2 and a monologue MAR greater than 7.5 produced a sensitivity of 0.92 and a specificity of 0.95.

**Conclusion:**

We conclude that combining monologue RDF and monologue MAR well-distinguished cluttering from stuttering. This method provides new objective diagnostic criteria, which can aid clinicians, therapists, and basic researchers.

## 1 Introduction

Cluttering is a speech disorder characterised by rapid and irregular speech. Because stuttering-like symptoms can also occur in cluttering, patients presenting with a chief complaint of speech disfluency may have a mixture of stuttering and cluttering (Van Zaalen-Op ‘t Hof et al., 2009; Miyamoto et al., 2007). In Japanese speakers, cluttering is not clearly defined (Miyamoto, 2011), and currently there is no straightforward, objective method to diagnose cluttering in Japanese speakers and to distinguish it from stuttering.

Stuttering is thought to arise from abnormal brain function. Thus, several studies of stuttering have sought to uncover a brain basis for the disorder (Etchell et al., 2018; Chang et al., 2019; Korosteleva et al., 2021). It has also been suggested that the underlying cause of cluttering is related to disruption of normal brain function (Seeman, 1970; Lebrun, 1996). However, more recent studies have begun to unravel its brain basis and to distinguish it from stuttering. Resting-state functional MRI suggests that people who clutter (PWC) are similar to fluent speakers, at least in terms of neocortical function (Ward et al., 2015), whereas people who stutter (PWS) are different from fluent speakers in terms of activity in certain cortical regions (Xuan et al., 2012; Shojaeilangari et al., 2021). Taken together, these findings suggest that cluttering and stuttering differ in terms of brain function and that the two disorders are distinct. Further complicating the diagnostic picture is that PWS and PWC may employ different approaches in managing their disfluent speech (Van Zaalen-Op ‘t Hof and Reichel, 2015). These differences between the two disorders imply that in order to effectively treat PWC, it is essential to be able to differentiate cluttering from stuttering in clinical practice. It is also imperative to be able to clearly distinguish the two for purposes of basic research.

The least common denominator (LCD) approach espoused by St Louis et al. (2007) is commonly used to define cluttering. The LCD approach states: “cluttering is a fluency disorder wherein segments of a conversation in the speaker’s native language typically are perceived as too fast overall, too irregular, or both” (St Louis et al., 2007). This common definition, however, is highly subjective, and there is little interprofessional agreement for diagnosing cluttering based on the subjective evaluation of speech symptoms (Van Zaalen-Op ‘t Hof et al., 2009). Moreover, such subjective diagnosis requires clinicians to have a high level of expertise and long-term experience with the two kinds of disfluent speech. Therefore, more objective diagnostic criteria are needed to distinguish cluttering from stuttering. Tools that use objective criteria could also be useful clinically to diagnose cluttering and potentially to treat it.

Van Zaalen-Op ‘t Hof et al. (2009) espouse using the ratio of disfluencies (RDF) as an objective measure. RDF is calculated by dividing the frequency of normal disfluencies (NDF) by the frequency of stuttering-like disfluencies (SDF). NDF is common in people who do not stutter, while SDF is common to PWS (Van Zaalen-Op ‘t Hof et al., 2009). RDF values of <1 indicate stuttering, whereas RDF values between 1 and 3 indicate cluttering-stuttering and RDF values of 3 or more indicate pure cluttering (Van Zaalen-Op ‘t Hof et al., 2009). However, this RDF criterion was developed and validated using Dutch speakers; thus, it is unclear whether it is applicable in other languages. In fact, when Iimura and Miyamoto (2021) applied the RDF criterion to adult Japanese speakers who stutter, they found that 89% of the PWS had some element of cluttering (56% cluttering, 33% cluttering-stuttering). This outcome was considered to be a case of overdiagnosis (Iimura and Miyamoto, 2021). They pointed out that this RDF approach is problematic for analyzing Japanese speech because interjectional expressions are used more frequently in normal spoken Japanese than in other languages, and these interjections could be mistaken as instances of cluttering. Interjections are expressions or abrupt remarks made especially as an aside or interruption. These are typically not full-fledged words (Goffman, 1978). They serve many functions in Japanese, and as a result, the NDF calculated from Japanese speech may have an artificially high value. Therefore, a multifaceted evaluation method to classify disfluency disorders is necessary for spoken Japanese (Iimura and Miyamoto, 2021) and perhaps other languages that employ more interjections in normal speech.

Against this background, new criteria are needed to differentiate cluttering from stuttering in Japanese speakers. The criteria must be objective and capture the characteristics of cluttering. Taking the LCD of cluttering into consideration again, “irregularity of speech rate” is difficult to measure objectively and cannot be used as an objective criterion. Thus, in the present study, we determined whether speech rate by itself could be used as a criterion. However, since speech rate is calculated as the number of words divided by speech duration, it is affected by the severity of dysfluency and other factors (Ochi et al., 2021). Thus, in the present study, we used articulation rate. Articulation rate is measured in the absence of disfluency symptoms and pauses. The RDF and mean articulation rate (MAR) under multiple conditions were calculated and tested to determine whether they could be useful for diagnosing cluttering.

## 2 Methods

### 2.1 Ethics statement

This study was approved by the ethics committee at Keio University School of Medicine (Authorization number: 20241077). It was designed and conducted according to the principles outlined in the Declaration of Helsinki (World Medical Association, 2013). Participants’ written informed consent was obtained before collecting data, and we employed procedures to protect participant privacy and anonymity.

### 2.2 Participants and speech data

Thirty-two patients who visited the Department of Otolaryngology, Head and Neck Surgery at Keio University Hospital served as participants. All patients who were seen between July 2020 and January 2023 with a primary complaint of dysfluent speech were included. No participant had an appropriate diagnosis other than stuttering or cluttering. All participants were native speakers of Japanese. Twelve (38%) of the 32 participants were diagnosed with cluttering according to the definition and criteria of St Louis et al. (2007). These criteria were applied to recordings of the participants’ recording of the Kitsuon Kensa-ho test (Ozawa et al., 2016). This test is commonly used to assess stuttering in Japanese speakers. The diagnosis was made by professional consensus of a physician and two speech-language-hearing therapists as they listened independently to patients’ Kitsuon kensa-ho recordings. None of the patients in either the cluttering or stuttering group had received specialised treatment for stuttering in the past.

Van Zaalen-Op ‘t Hof et al. (2009) observed a third category of patients who present with both cluttering and stuttering symptoms and coined the term “cluttering-stuttering” to describe them. We did not adopt this “cluttering-stuttering” concept in the present study because it was considered to be uncommon, being an original classification of Van Zaalen-Op ‘t Hof et al. (2009). Therefore, for the present study, we defined cluttering to include both of what Van Zaalen-Op ‘t Hof et al. classified as cluttering-stuttering and cluttering.

### 2.3 Ratio of disfluencies (RDF)

The RDF was calculated from scores on the Kitsuon Kensa-ho recordings. Among the tasks of the Kitsuon Kensa-ho test, the “oral reading” and “monologue” portions included sufficient amounts of speech to analyze for our purposes.

SDF and NDF were also calculated from speech data obtained from the Kitsuon Kensa-ho recordings. We used the definitions of Van Zaalen-Op ‘t Hof et al. (2009) to obtain the SDF and NDF. SDF are “tense word repetition,” “tense part-word repetition,” “prolongation,” and “block.” NDF are “word repetition,” “part-word repetition,” “interjection,” “revision,” and “phrase repetition.” The percentage of *bunsetsu* in which SDF and NDF occurred was also calculated. A *bunsetsu* is a linguistic unit in Japanese that is as long as, or longer than, a word but smaller than a phrase. In Japanese, disfluency is generally evaluated in terms of *bunsetsu* (LaSalle and Huffman, 2015; Iimura and Miyamoto, 2021; Ozawa et al., 2016).

To ensure the reliability of the SDF and NDF data, 25% of randomly selected *bunsetsu* were evaluated by a second rater. Inter-correlation coefficients of the raters were 0.81 [0.56–0.99 (95% confidence interval)] for the SDF and 0.96 [0.80–0.99] for the NDF. These were calculated based on a single rater, absolute agreement, and 2-way mixed-effects model (Koo and Li, 2016).

For each task, the RDF was calculated as the ratio of NDF to SDF (NDF:SDF). As Iimura and Miyamoto (2021) observed, some cases do not have a measurable SDF. Since the RDF could not be calculated in those cases, we considered the RDF in these cases to be a sufficiently large number, and thus assigned it a value of 10 (one case in RDF on the oral reading and two cases in RDF on the monologue).

### 2.4 Mean articulatory rate (MAR)

From patients’ recordings of the oral reading and monologue tasks of the Kitsuon Kensa-ho, we randomly selected three parts that had the following characteristics: (1) 8 to 20 morae, (2) SDF absent, and (3) 250 msec or longer of speech without pauses (Van Zaalen-Op ‘t Hof and Reichel, 2015). Next, we calculated the speed of articulation by dividing the number of morae by duration, and we averaged the speed of articulation of these three parts to obtain the average articulation speed.

Praat speech analysis software (Boersma and Weenink, 2024) was used to measure these parameters. This software can analyze various acoustic characteristics of the recordings, like sound intensity, pitch amplitude, and duration or formants. The acoustic spectrograms of the speech recordings comprised the input data for the software. The starting point of pronunciation in a spectrogram was taken as the point at which the fundamental or formant frequency appeared for vowels. For consonants, depending on the type, the starting point of pronunciation was taken as the point at which the consonant component (e.g., the burst part for bursts) could be identified. Since Japanese is a language in which words end with a vowel (including formant frequencies), the end point was defined as the point where the formant, or fundamental frequency disappeared from the sound spectrogram.

To ensure the reliability of MAR, 25% of the randomly selected *bunsetsu* were evaluated by a second rater, whose inter-correlation coefficient was 0.91 [0.83–0.95 (95% confidence interval)]. These were calculated based on a single rater, absolute agreement, and 2-way mixed-effects model (Koo and Li, 2016).

### 2.5 Statistical analysis and setting cutoff values

SPSS 26 was used for statistical analyses. Differences between cluttering and stuttering groups in the RDF and MAR for the oral reading and monologue data were evaluated by the Mann–Whitney U test. The significance level was set at *p* < 0.05.

Receiver operating characteristic curves (ROC) were constructed (Nahm, 2022) and used to select the optimal cutoff points to distinguish groups. In this analysis, we aimed to assess performance of the RDF and MAR parameters as a diagnostic tool over the range of possible cutoff points. ROC curves were made separately for the RDF and MAR data for the oral reading and monologue (Mandrekar, 2010). The earlier diagnosis by an expert was considered to be the gold standard in the ROC analyses. This is the disorder status for each patient measured without error. We identified points on the ROC curve that were the smallest distance from the point where both sensitivity and specificity had values of 1 (i.e., the point where $\sqrt{{\left(1-\mathrm{sensitivity}\right)}^{2}+{\left(1-\mathrm{specificity}\right)}^{2}}$ is the minimum) and calculated the sensitivity and specificity at these points (Nahm, 2022). The most appropriate combination of items for diagnosis was examined in terms of calculated sensitivity and specificity.

## 3 Results

The cluttering and stuttering groups did not differ significantly in age or gender (Table 1).

**Table 1:** Characteristics of participants.

	Stuttering group	Cluttering group	*p*-value
*N*	20	12	
Sex ratio (male:female)	18:2	11:1	0.50^a^
Age (y) Median (Range)	24 [18–42]	23.5 [18–45]	0.82^b^

^aFisher’s exact test; bMann–Whitney U test.^

We first compared the cluttering and stuttering groups for differences in the RDF and the MAR parameters on the Kitsuon Kensa-ho test (Table 2). There were no significant differences between the cluttering and stuttering groups on either the RDF or MAR parameters on the oral reading part of the Kitsuon Kensa-ho test (Table 2). However, the cluttering and stuttering groups had significantly different MAR values on the monologue part of the Kitsuon Kensa-ho test; the MAR of the cluttering group was significantly larger than that of the cluttering group (8.7 vs. 7.0; *p* = 0.001).

**Table 2:** Group comparison of RDF and MAR parameters on oral reading and monologue parts of the Kitsuon Kensa-ho test.

	Stuttering group (median [range])	Cluttering group (median [range])	*P*-value^a^
RDF of oral reading	0.18 [0.0–10]	0.41 [0.0–10]	0.31
RDF of monologue	1.4 [0.038–10]	3.0 [1.1–26]	0.064
MAR of oral reading	7.0 [5.4–9.4]	7.5 [5.7–8.9]	0.14
MAR of monologue	7.0 [4.9–8.6]	8.7 [5.5–10]	0.001

RDF, Ratio of disfluencies; MAR, mean articulatory rate. ^a^Mann–Whitney *U* test.

**Figure 1:**
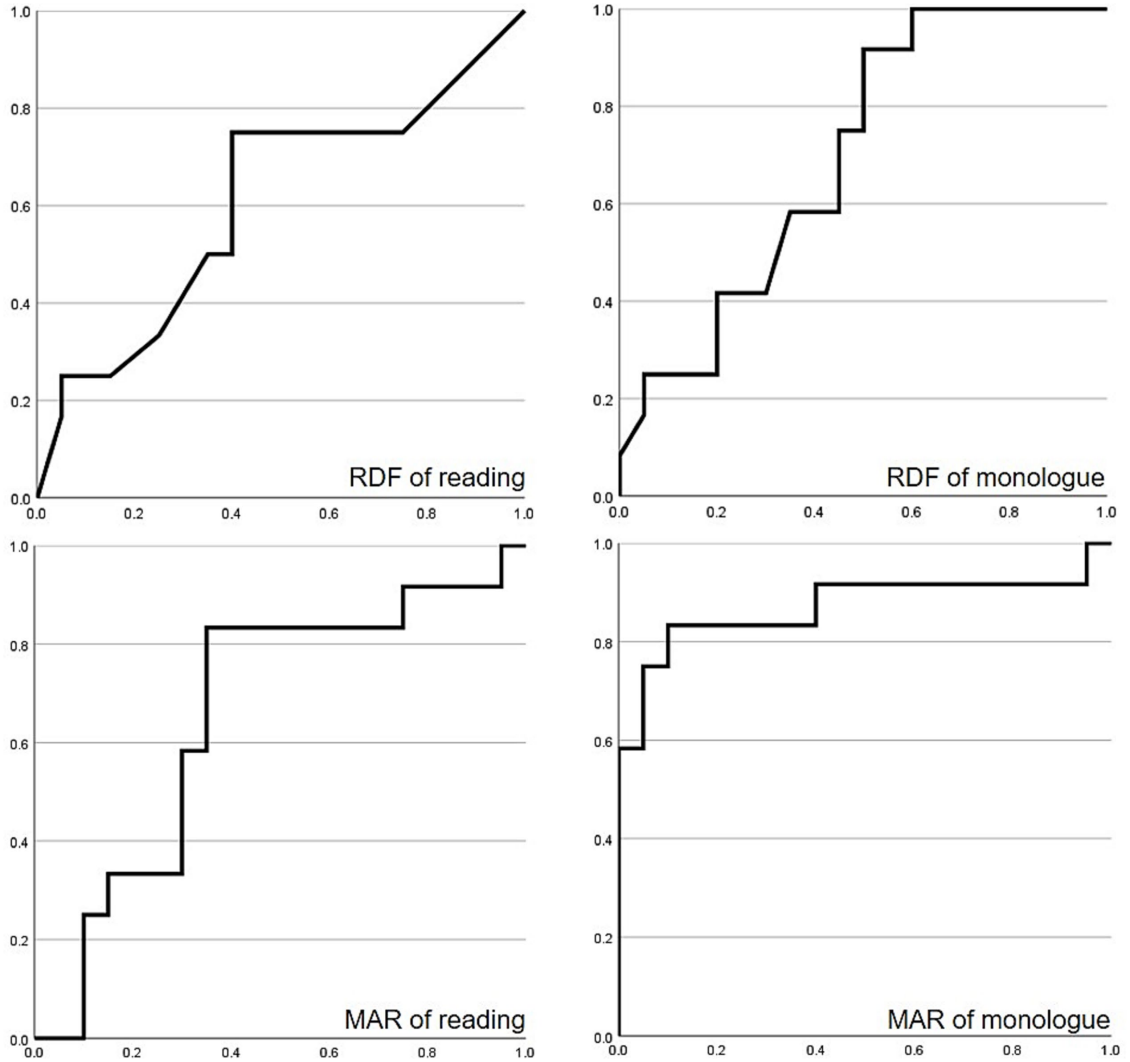
Receiver operating characteristic curves for RDF and MAR data obtained from participants with dysfluent speech. Considering the diagnosis of cluttering to be positive (i.e., the gold standard), we generated separate sensitivity and specificity receiver operating characteristic (ROC) curves for the ratio of dysfluencies (RDF) and mean articulation rate (MAR) of the oral reading and monologue parts of the Kitsuon Kensa-ho test. Abscissa on each graph represents 1 - specificity and the ordinates represent sensitivity. Data shown from 32 participants with dysfluent speech.

ROC curves were created separately for the RDF and MAR results for the oral reading and monologue parts of the Kitsuon Kensa-ho test (Figure 1). Considering the cluttering diagnosis to be a positive diagnosis, we generated sensitivity and specificity graphs for RDF and MAR of the oral reading and monologue data. The area under the curve (AUC) was 0.61 for the RDF of oral reading, 0.70 for the RDF of monologue, 0.66 for the MAR of oral reading, and 0.87 for the MAR of monologue (Table 3). The larger the AUC, the more useful the test is for differential diagnosis. The results of the MAR monologue data had the largest AUC.

**Table 3:** Area under the curve for RDF and MAR of oral reading and monologue and cutoff values.

	AUC	Cutoff value	Sensitivity	Specificity
RDF of oral reading	0.61	0.30	0.75	0.60
RDF of monologue	0.70	1.2	0.92	0.50
MAR of oral reading	0.66	7.1	0.83	0.65
MAR of monologue	0.87	7.5	0.83	0.90

AUC, Area under the curve; RDF, ratio of dysfluencies; MAR, mean articulation rate.

To set cutoff values, we first identified the points on the ROC curve closest to the point where sensitivity and specificity both equaled one and then calculated the sensitivity and specificity at these points. This represented the cutoff values for the given test parameter. The cutoff values (sensitivity and specificity) for the RDF of the oral reading were 0.30 (0.75, 0.60); for the RDF of the monologue task, they were 1.2 (0.92, 0.50); for the MAR of the oral reading, they were 7.1 (0.83, 0.65); and for the MAR of the monologue task, they were 7.5 (0.83, 0.90) (Table 3). The RDF of the monologue task had the best sensitivity, whereas the MAR of monologue task had the best specificity. For subjects who had no SDF, we set the RDF to 10 (one case in RDF on the oral reading and two cases in RDF on the monologue). Even when data for these non-SDF subjects were excluded, the cutoff was not affected. For this “exclusion test,” the cutoff values for the RDF of oral reading were 0.30 (0.73, 0.6), and the cutoff values for the RDF of the monologue task were 1.2 (0.91, 0.5).

To increase the diagnostic accuracy of these tests for cluttering, we assessed the sensitivity and specificity of combinations of two or more items of the Kitsuon Kensa-ho test. Combining multiple items did increase the accuracy of the cluttering diagnosis. An RDF for the monologue task >1.2 together with a MAR for the monologue task >7.5 showed superior sensitivity (0.92) and specificity (0.95) as diagnostic criteria for cluttering.

## 4 Discussion

In the present study, we established differential diagnostic criteria in an effort to more clearly distinguish cluttering from stuttering in disfluent oral speech of Japanese speakers. The two disorders are thought to share some overlap of symptoms (Van Zaalen-Op ‘t Hof et al., 2009; Miyamoto et al., 2007), and thus, it has been difficult to clearly distinguish them. The differential diagnostic criteria we established here used quantitative differences in spoken speech in PWS and PWC.

ROC analysis of the calculated RDF and MAR data for two oral subtests of the Kitsuon Kensa-ho test (Ozawa et al., 2016), “oral reading” and “monologue,” revealed that the RDF for the monologue was the most sensitive for distinguishing cluttering from stuttering. The MAR for the monologue was the most specific. Therefore, the two were combined to establish a provisional diagnostic criterion for cluttering. With this criterion, the RDF for the monologue is >1.2 and the MAR for the monologue is >7.5. This criterion has a sensitivity of 0.92 and a specificity of 0.95, indicating it has excellent diagnostic accuracy. The subjective diagnosis of cluttering has a low rate of inter-rater agreement and reproducibility, as it is difficult for speech experts to agree on what constitutes cluttering (Van Zaalen-Op ‘t Hof et al., 2009). By contrast, the objective criteria established in the present study showed a high rate of inter-rater agreement and diagnostic reproducibility.

In the present study, patients were first evaluated for cluttering by a medical doctor and speech-language-hearing therapists using the criteria of St Louis et al. (2007). A diagnosis of cluttering was made based on a consensus of their findings, which is an alternative to the gold standard for diagnosis. In this evaluation, 12 of 32 patients (38%) who presented with a chief complaint of language disfluency were diagnosed with cluttering. In a situation similar to the present study, Van Zaalen-Op ‘t Hof et al. (2009) evaluated adult speakers of Dutch presenting with a chief complaint of disfluency. They diagnosed 61% of their participants to have a “component of cluttering.” Among Japanese speakers, a study of elementary school students found that 15.9% of the children attending a day class had cluttering (Miyamoto et al., 2007). Although the percentages vary from study to study, the cluttering percentage of 38% in the present study is reasonable.

Van Zaalen-Op ‘t Hof et al. (2009) proposed that when a patient’s RDF is 1.0 or higher, a diagnosis of cluttering can be made. In the present study, we computed an RDF cutoff value of 1.2, which is close to Van Zaalen’s-Op ‘t Hof et al.’s cluttering criterion. However, if Van Zaalen’s-Op ‘t Hof et al.’s criterion is applied to Japanese speakers, more patients are misdiagnosed or overdiagnosed with cluttering (Iimura and Miyamoto, 2021). Although our RDF cutoff value is greater than that of Van Zaalen-Op ‘t Hof et al., its specificity is rather low, having a value of 0.5. This means that using only one criterion — the RDF value — as a basis for diagnosing cluttering would lead to overdiagnosis. Thus, to diagnose cluttering more accurately, a combination of diagnostic criteria should be used.

Here, we proposed that MAR, a measure of articulation rate, should be included in the criteria for diagnosing cluttering in Japanese speakers. We assessed the MAR of the monologue subpart in the Kitsuon Kensa-ho test to differentiate cluttering with the highest accuracy among the items tested in this study. Nonetheless, using only the MAR for the monologue to differentiate cluttering resulted in a sensitivity of only 0.83. Therefore, to boost test sensitivity, we decided to combine the RDF for the monologue (RDF > 1.2) and the MAR for the monologue (MAR > 7.5) as diagnostic criteria for identifying cluttering. Together they have a sensitivity of 0.92 and specificity of 0.95, indicating that, when combined together, they have excellent accuracy for diagnosing cluttering.

The MAR and RDF data we used in the criteria for stuttering-cluttering differentiation were monologue speech data, not the oral reading data. The basis of symptoms of cluttering lies in a problem with language planning (St Louis et al., 2003; Van Zaalen-Op ‘t Hof et al., 2009; Van Zaalen-Op ‘t Hof and Reichel, 2015; Georgieva, 2020). Since oral reading does not require planning—such as word recall or sentence construction—we reasoned that having participants’ oral reading data would not reveal cluttering symptoms. In previous studies of a different language (Dutch) (Van Zaalen-Op ‘t Hof et al., 2009), RDF and MAR did not differ between the stuttering and cluttering groups in oral reading. These results suggest that oral reading is not useful for differential diagnosis, because it does not reveal differences that would help in differentiating stuttering from cluttering. Nevertheless, oral reading is a useful task that allows for comparison and evaluation without being affected by the amount or content of speech, as it involves reading the same sentences. Various speech characteristics can be evaluated with oral reading tasks. For example, one speech characteristic affected in cluttering is intonation. PWC exhibit little intonation (Daly and Burnett, 1999; Van Zaalen-Op ‘t Hof and Reichel, 2015). However, as these evaluation items are difficult to quantify, they were not included in the present study. To achieve a more multifaceted diagnosis of cluttering, it would be beneficial to consider incorporating them in future studies.

Despite the utility of our two-item criterion for objectively diagnosing cluttering, it does have some limitations. One is that speech rate has traditionally not been considered to be useful for diagnosing cluttering. While rapid speech rate is considered to be a characteristic of cluttering, some have reported speech rate in people who clutter to be within the normal range when it is measured objectively (Levelt, 1989; Bakker et al., 2011). Similarly, the speech rate of Japanese speakers who clutter is reported to be slower than previously thought (Miyamoto, 2019; Iimura and Miyamoto, 2021). One possible reason for this apparent contradiction to our present results is that we compared the articulation rate of patients with presumed cluttering with those who stuttered (disfluent, non-cluttering) rather than with those who were fluent. The articulation speed of adults who stutter is generally slow (Meyers and Freeman, 1985; Ochi et al., 2021), while the articulation speed of people who clutter is comparable to that of fluent people and faster than that of people who stutter (Van Zaalen-Op ‘t Hof et al., 2009). Therefore, although articulation rate is not useful for differentiating cluttering from fluent speech, it is useful for differentiating cluttering from stuttering. However, to the best of our knowledge, no previous research has been done to assess the articulation rate of fluent Japanese speakers. Thus, we cannot say for certain whether that articulation rate of Japanese PWC is comparable to that of fluent Japanese speakers and whether it can be used as a diagnostic criterion. Since we did not obtain data on fluent Japanese speakers in the present study, the data we obtained on the articulation rate of PWC in this study can be considered preliminary.

Another possible reason is that there may be differences in the population of subjects in previous studies and our subjects, particularly subject age. The previous study focused mainly on teenagers (Van Zaalen-Op ‘t Hof et al., 2009). It is reported that articulation rate generally increases between the ages of 11 and 21 (Van Zaalen-Op ‘t Hof and Reichel, 2015) Thus, it may be difficult to characterise cluttering based on articulation rate alone in the group of teenagers. In the present study, the subjects were all 18 years of age or older, making it easier to detect differences in articulation rate between non-cluttering (including stuttering) and cluttering.

A second limitation is the problem of classifying dysfluent symptoms. In particular, it was impossible to distinguish objectively between “tense word repetition” and “word repetition” and between “tense part-word repetition” and “part-word repetition.” No method to distinguish between them has been clearly demonstrated in previous studies (Van Zaalen-Op ‘t Hof et al., 2009). One criterion used was the volume of speech immediately before the onset of symptoms and the volume of speech of the dysfluency symptom, but this would have been a subjective method. Another factor may have been that the present study was based on speech-only recordings (pure audio data). Using quantitative video recordings could have helped us to differentiate cluttering and stuttering by assessing participants’ muscle tone. Our clinical setting, however, precluded this possibility. However, the intraclass correlation coefficient (ICC) between the raters in the present study was large enough not to have a significant impact on the final differential diagnosis.

A third limitation of the present study is that it did not use self-report measures of symptoms. The use of self-reports for stuttering symptoms has been reported to be an effective method for evaluating and treating stuttering (Tichenor and Yaruss, 2019; Boyce et al., 2022; Tichenor et al., 2022; Herring and Yaruss, 2022). Because therapists can observe only some symptoms, self-reports can be useful to gain and thus evaluate the overall experience of communication. As mentioned above, “tension” is difficult to evaluate. However, self-reports by people with stuttering have been reported to be useful for evaluating “tension” (Tichenor and Yaruss, 2019). From this observation, it is possible that self-reporting of symptoms could also be useful for diagnosing cluttering. However, there is no clear evidence yet on whether self-reporting is indeed useful for diagnosing cluttering. Therefore, we reasoned that it would be more useful to use LCD, as many studies have diagnosed cluttering based on LCD assessments (Scott, 2020; Ward et al., 2015). It is necessary to discuss in the future what kind of self-reporting is specific to cluttering and whether it is useful for diagnosing cluttering.

A fourth limitation is that the present study was conducted in a retrospective manner. Although it was possible to distinguish cluttering from stuttering with a high degree of accuracy in the population we studied, conducting a prospective study would bolster reproducibility.

## 5 Conclusion

We established an objective method of differentiating cluttering from stuttering in Japanese speakers. By combining two criteria (RDF for monologue >1.2 and MAR for monologue >7.5) to differentiate cluttering from stuttering in people presenting with dysfluencies, we were able establish a new diagnostic criterion having high sensitivity and specificity for cluttering. This objective diagnostic criterion may be able to aid clinicians, therapists, and basic researchers to distinguish cluttering from stuttering.

## Data Availability

The raw data supporting the conclusions of this article will be made available by the authors, without undue reservation.
